# Untargeted metabolomics analysis of *Physalis pubescens L.* with respect to different varieties

**DOI:** 10.3389/fnut.2025.1629774

**Published:** 2025-11-18

**Authors:** Song Yan, Jialei Li, Kaixin Chen, Shan Shan, Shan Zhang, Yang Gao, Bin Liu

**Affiliations:** 1 Food Processing Research Institute, Heilongjiang Academy of Agricultural Sciences, Harbin, China; 2 Pharmaceutical Engineering Technology Research Center, Harbin University of Commerce, Harbin, China

**Keywords:** *P. pubescens*, LC-MS, untargeted metabolomics, differential metabolites, metabolic pathways

## Abstract

*Physalis pubescens L.* is a nutritious fruit with recognized pharmacological value, yet its comprehensive metabolomic profile remains unexplored. This study aimed to investigate the metabolomic differences among three distinct varieties of *P*. *pubescens*, with a focus on the influence of fruit size. An untargeted metabolomics approach employing UPLC-ESI-MS/MS was utilized. Multivariate statistical analyses, including PCA and PLS-DA, revealed a clear separation in metabolic profiles, primarily driven by fruit size. Comparative analysis between large-fruited variety B and small-fruited varieties S and T identified 67 significant differential metabolites. Notably, the flavonoid quercetin was not detected in large-fruited variety B under our analytical conditions, and the relative content of most phenylpropanoid metabolites was significantly lower in large-fruited variety compared to small-fruited varieties. Conversely, 17 metabolites, including certain amino acids and riboflavin, were up-regulated in large-fruited variety. Pathway analysis highlighted riboflavin metabolism as a key distinguishing pathway. Our findings demonstrate that fruit size may be a major factor influencing the phytochemical composition of *P. pubescens*. The novelty of this work lies in establishing fruit size as a major factor shaping the phytochemical composition of *P. pubescens*. These findings provide a metabolic foundation for selecting varieties with desired nutraceutical properties and for guiding future quality control and breeding programs.

## 1 Introduction

*Physalis pubescens L.*, also known as “huang gu niang”, is an annual herb in the Solanaceae family, and its ripe fruits are rounded and golden yellow (1, 2). It is native to South America and is wild and cultivated in northeastern China. *P. pubescens* has a variety of pharmacological uses and its fruits, calyxes, branches, and leaves have a wide range of medicinal activities. Studies have shown that it has high edible and medicinal value, containing 18 essential amino acids, 21 trace elements, as well as a large number of minerals, vitamins, and unsaturated fatty acids (3). It contains steroids, flavonoids, and other chemical compounds. The holistic nutritional effects of *P. pubescens* are largely attributed to its phytochemicals, which have been used in the treatment of coughs, phlegm, pharyngitis, sore throats, and urinary difficulties (4, 5). It is also widely used to treat diabetes and various skin diseases. In addition, *P. pubescens* has been found to exhibit good anti-tumor activity (6). Recent studies have highlighted the significant impact of growing conditions and genetic background on the bioactive compound profiles in different physalis species (7). Furthermore, the antioxidant and neuroprotective potential of *P. pubescens* polysaccharides has been confirmed, underscoring the value of in-depth exploration of its metabolites (8). *P. pubescens* may contain a large number of unknown active ingredients that may play an important role in disease prevention and treatment. However, the identification of potential markers is challenging and requiring new methods to find accurate biomarkers.

Currently, most research on *P. pubescens* focuses on its chemical composition and fruit processing. However, the metabolic profiles and related pathways within the fruit have received little attention. This lack of knowledge has led to unclear quality attributes of *P. pubescens* raw materials, which in turn has hindered the development and application of its health products. At present, HPLC-DAD-ESI-MS and HR-ESI-MS were applied to preliminarily identify 18 compounds from hydromethanolic extract of physalis fruits. Diverse mono and dihexosides of cinnamic, coumaric, caffeic, ferulic, and sinapic acids, and also N, N′-dicaffeoylspermidine isomers were found in *P. pubescens*, in addition, two HDMF (4-hydroxy-2,5-dimethyl-3(2 h)-furanone) hexosides were identified for the first time from *P. pubescens* (9). Untargeted Metabolomics detects all small molecule metabolites in biological samples such as cells, tissues, organs or body fluids without bias and in as many ways as possible, and is widely used in various applications (10, 11). Differential metabolites are screened by statistical analysis, and metabolic pathway analyses are performed on the differential metabolites, thus looking for the relative relationship between the metabolites and physiological and pathological changes. Plant metabolomics contributes to the understanding of the underlying mechanisms of plant metabolite responses to environmental changes or genetic mutations. Liquid chromatography-mass spectrometry (LC-MS) is a method that can be used for the detection of metabolites with high resolution and sensitivity. Currently, metabolomics has been used to study the effect of different factors such as environmental factors (12), developmental stages (13), season (14), and variety (12, 15) on the chemical composition and quality of plants.

Despite these valuable findings, current research on *P. pubescens* has predominantly focused on either the identification of specific compound classes or fruit processing. A significant knowledge gap remains in our comprehensive understanding of its global metabolic profile and, more importantly, how the metabolome varies systematically among different varieties, particularly those with distinct morphological traits such as fruit size and shape. This lack of a holistic metabolic perspective has hindered the establishment of objective quality standards and the targeted development of health products based on specific phytochemical compositions. To bridge this gap, we turned to untargeted metabolomics, a powerful and novel approach that provides an unbiased, comprehensive snapshot of the small molecule complement within a biological system. Unlike targeted methods, this strategy is ideally suited for the simultaneous discovery of both known and novel biomarkers. In this study, we employed UPLC-ESI-MS/MS-based untargeted metabolomics to investigate and compare the metabolic profiles of three distinct varieties of *P. pubescens* that exhibit clear differences in fruit morphology. The primary objectives of this study were to: determine whether the metabolic profiles of these three varieties are statistically distinct; identify and characterize the key differential metabolites that contribute to these distinctions, with a special focus on the comparison between large and small fruits; and elucidate the potential metabolic pathways involved. This work provides a foundational metabolic dataset that is expected to enhance the utilization, quality control, and variety breeding of *P. pubescens*.

## 2 Materials and methods

### 2.1 Plant materials and sample preparation

*Physalis pubescens*: Three varieties of *P. pubescens* were investigated: “yi ke song” (S variety) planted in Muling City, Heilongjiang Province, “tie ba qing” (T variety) planted in Muling City, Heilongjiang Province (GPS: 44.9106° N, 130.5258° E; altitude: ~250 m), and “bing he” (B variety) planted in Qinggang County, Harbin City, Heilongjiang Province (GPS: 46.6846° N, 126.1058° E; altitude: ~180 m). The fruits were harvested in early August 2023. For each variety, three independent biological replicates were prepared. Each biological replicate was composed of three whole fruits randomly sampled from different individual plants within the respective cultivation plot. After harvesting, each replicate (three fruits) was sealed in a separate 50 mL centrifuge tube. The samples were then processed independently: the persistent calyx was removed, they were wrapped in tin foil, quick-frozen in liquid nitrogen, and stored in cryovials at −80 °C for subsequent metabolite extraction. This experimental design with three biological replicates per variety allowed us to account for plant-to-plant biological variation and to perform robust statistical analyses of the metabolomic data.

### 2.2 Chemicals and reagents

HPLC-MS grade methanol, acetonitrile, acetic acid were purchased from ANPEL. Laboratory Technologies (Shanghai, China) Inc. Ultrapure water was prepared using a Milli-Q water purification system (Millipore, USA).

### 2.3 Morphological characterization

The length and width of the physalis fruits were measured via electronic vernier calipers (Shanghai Measuring Instrument Co., Shanghai, China).

### 2.4 Untargeted metabolomics analysis

#### 2.4.1 Metabolite extraction

Frozen fruit samples (approximately 100 mg) were ground in liquid nitrogen. The metabolites were extracted with 0.5 mL of 80% aqueous methanol using ultrasonication for 30 min, followed by centrifugation at 12,000 rpm for 10 min at 4 °C. The supernatant was collected. This extraction procedure was repeated three times, and the combined supernatants were pooled for subsequent analysis (12, 15).

#### 2.4.2 UPLC-ESI-MS/MS analysis

Chromatographic separation was performed on a Thermo Vanquish UPLC system (Thermo Scientific, USA) equipped with a Waters HSS T3 column (50 × 2.1 mm, 1.8 μm). The column temperature was maintained at 40 °C. The mobile phase consisted of (A) water with 0.1% acetic acid and (B) acetonitrile with 0.1% acetic acid. The gradient elution program was as follows: 0–2.0 min, 10% B; 2.0–6.0 min, 10%–60% B; 6.0–8.0 min, 60% B; 8.0–8.1 min, 60%–10% B; 8.1–12.0 min, 10% B for re-equilibration. The flow rate was 0.3 mL/min, and the injection volume was 2 μL. All samples were maintained at 4 °C in the autosampler during the analysis. To monitor system stability and data reliability, quality control (QC) samples were prepared by pooling aliquots from all samples and were injected at regular intervals throughout the analytical sequence (9, 16).

#### 2.4.3 Mass spectrometry conditions

MS data were acquired using a Q Exactive hybrid quadrupole-Orbitrap mass spectrometer (Thermo Scientific) equipped with a heated electrospray ionization (HESI) source. The instrument was operated in both positive and negative ionization modes with a full MS-ddMS2 data-dependent acquisition strategy. The key source parameters were set as follows: spray voltage, ±2.8 kV; sheath gas pressure, 40 arb; aux gas pressure, 10 arb; capillary temperature, 320 °C; and heater temperature, 350 °C The full scan range was set to m/z 100–900 with a resolution of 70,000.

#### 2.4.4 Data processing and multivariate statistical analysis

The raw data files were converted from .raw format to .mzML format using Xcalibur 4.1 software (Thermo Scientific). Data preprocessing, including peak picking, alignment, and integration, was performed using the XCMS package in R (17), resulting in a data matrix of retention time, m/z, and peak intensity. Metabolite identification was conducted by matching the accurate mass and MS/MS spectra against the HMDB and METLIN databases.

For multivariate statistical analysis, the data matrix was normalized to the total peak area and then imported into SIMCA-P+ 14.0 (Umetrics, Sweden). Both unsupervised Principal Component Analysis (PCA) and supervised Partial Least Squares-Discriminant Analysis (PLS-DA) were performed. The model quality was assessed by the parameters *R*^2^*X* (or *R*^2^*Y*) and *Q*^2^, representing the explained variance and predictive ability, respectively (13). A permutation test (*n* = 200) was conducted to validate the PLS-DA model and prevent overfitting.

Differential metabolites between groups were selected based on a combination of Variable Importance in Projection (VIP) scores from the PLS-DA model (VIP > 1.0) and fold change (FC) thresholds (FC ≥ 2.0 or FC ≤ 0.5) (12). Finally, pathway enrichment analysis of the differential metabolites was performed using the Kyoto Encyclopedia of Genes and Genomes (KEGG) database.

## 3 Results

### 3.1 Morphological characterization of the three varieties of *Physalis pubescens*

**Figure 1:**
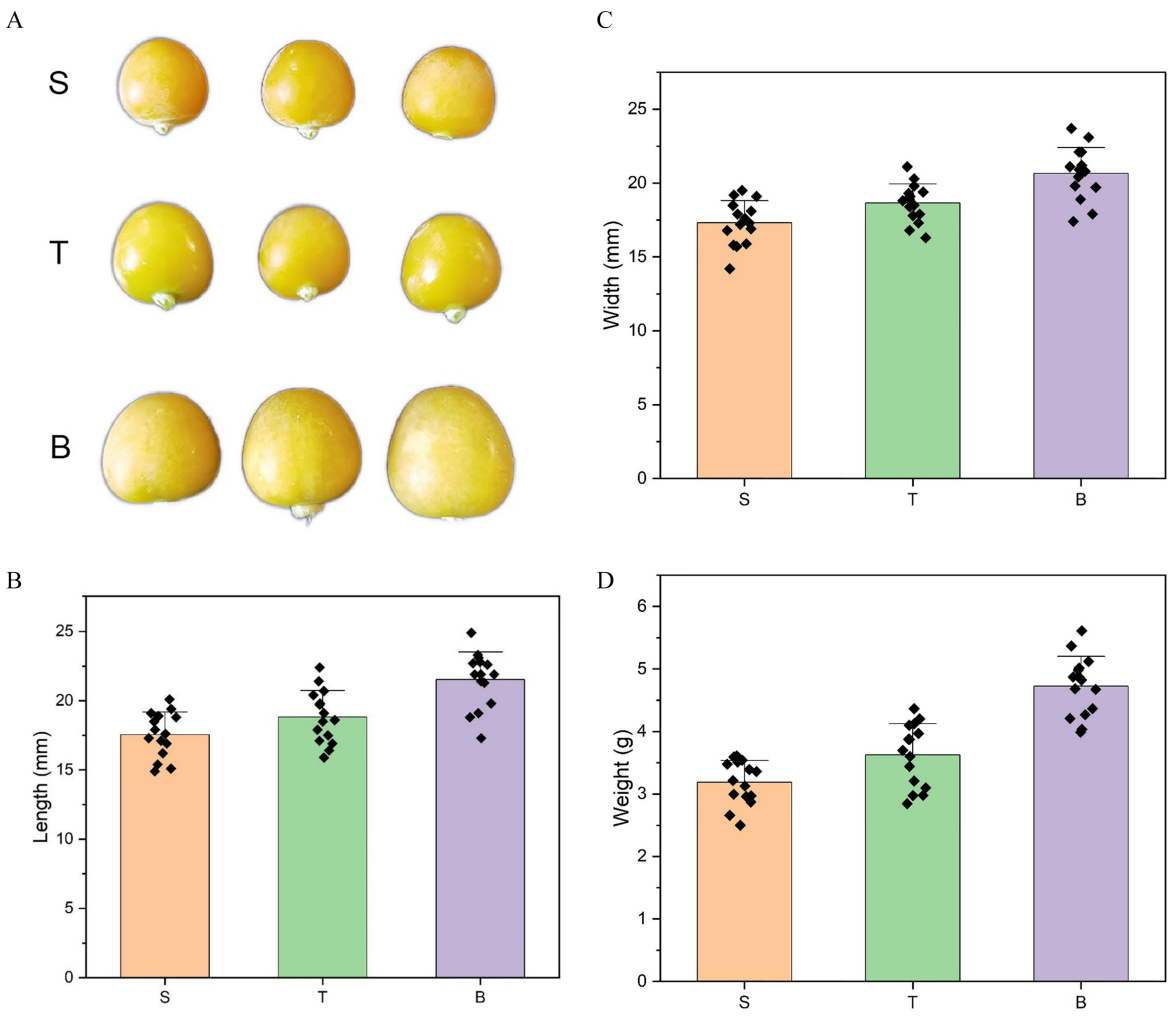
The appearance **(A)**, length **(B)**, width **(C)**, and weight **(D)** of the three varieties of *P. pubescens*. S, T, and B represent the varieties “yi ke song”, “tie ba qing”, and “bing he”, respectively. The data are presented as the mean ± standard deviation (SD).

Figure 1 presents the appearance of the three varieties of *P. pubescens*, as well as the length, width and weight of the individual *P. pubescens*. The mean lengths of the *P. pubescens* of S variety, T variety and B variety were 17.55 ± 1.57 mm, 18.82 ± 1.85 mm, and 21.52 ± 1.93 mm, respectively. The length of the B variety was significantly greater than those of S variety and T variety by 22.62% and 14.35%, respectively (*p* < 0.001 and *p* < 0.001, respectively). There was no significant difference between the lengths of S variety and T variety (*p* = 0.059) (Figure 1B). In terms of width, B variety had the greatest physalis fruit width, and the S variety had the smallest physalis fruit width. There was significant difference in width between S variety and T variety (*p* = 0.014, which is below the significance threshold of *p* < 0.05). The width of the B variety was significantly more than those of S and T (*p* < 0.001 and *p* < 0.001, respectively) (Figure 1C). Therefore, variety S and variety T are called small-fruited varieties, variety B are called large-fruited variety. The weight is one of the morphological indicators of physalis, and there were significant differences in the weight among large-fruited variety and small-fruited varieties (*p* < 0.001) (Figure 1D).

### 3.2 QC analysis

**Figure 2:**
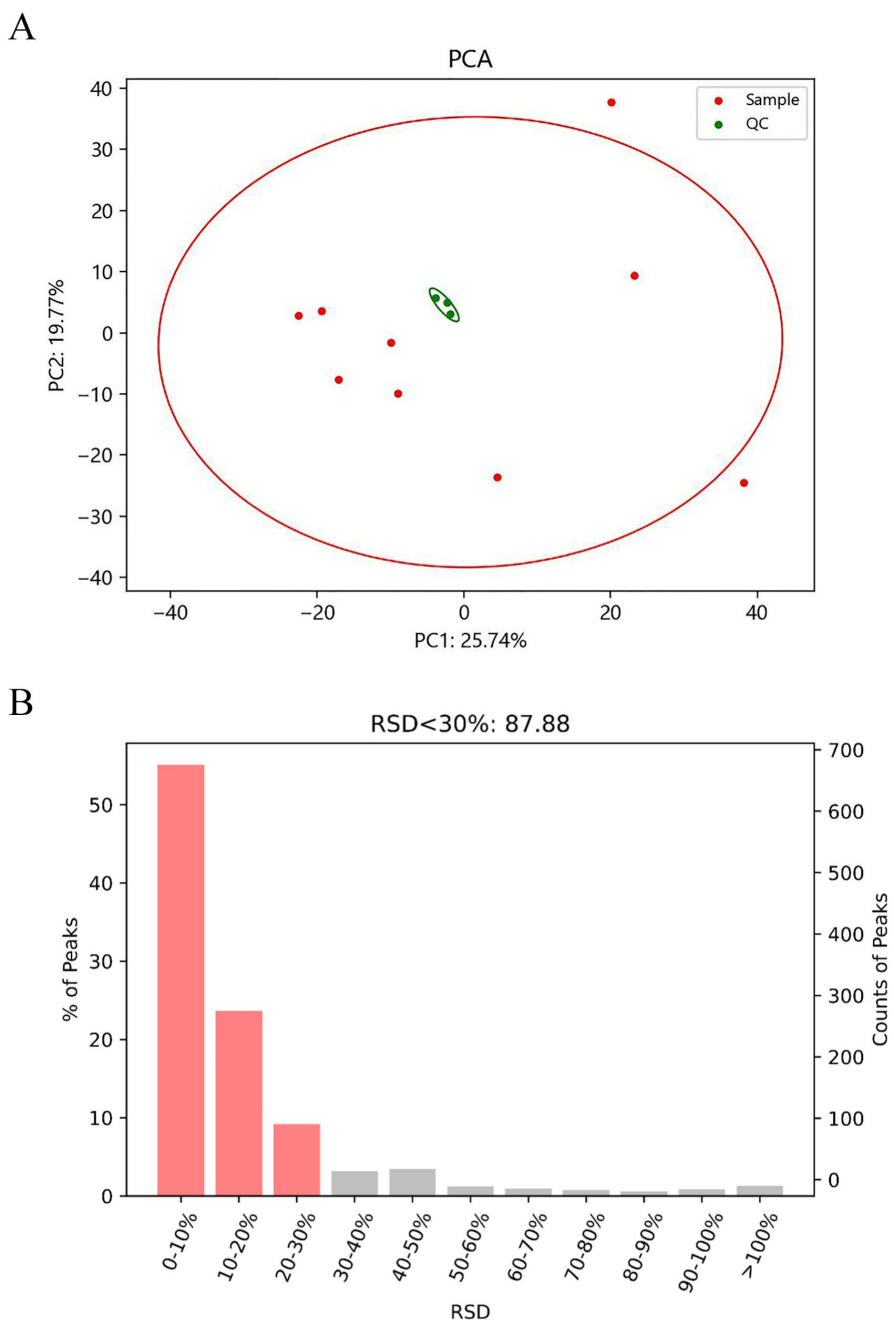
PCA score plot of raw detection data **(A)**. The smaller the variation between QC samples, the higher the method stability and the better the data quality. When visualized in a PCA score plot, this is represented by QC samples being more tightly clustered together; Histogram of RSD distribution of raw detection data **(B)**. The RSD (Relative Standard Deviation) of QC samples, which is the ratio of the standard deviation to the mean, is used to evaluate the stability and reliability of metabolite detection. A smaller RSD value indicates more stable and reliable detection results.

Quality control is required to obtain reliable and high quality metabolomics data. The QC samples were used for quality control during testing, and the QC samples were all consistent, but systematic errors would be generated during sample extraction, detection and analysis, The denser the distribution and the smaller the difference of QC samples indicated the higher the stability of the method and the better the quality of the data (Figure 2A). The RSD (Relative Standard Deviation) of QC samples, i.e., the ratio of standard deviation to the mean (Figure 2B), was also used to evaluate the stability and reliability of the metabolite assay, and the smaller the RSD value represented the more stable and reliable results. According to the histogram of RSD distribution, metabolites with RSD < 30% were selected for subsequent analysis.

### 3.3 Differences in the metabolomes of different varieties of *Physalis pubescens*

#### 3.3.1 Principal component analysis

**Figure 3:**
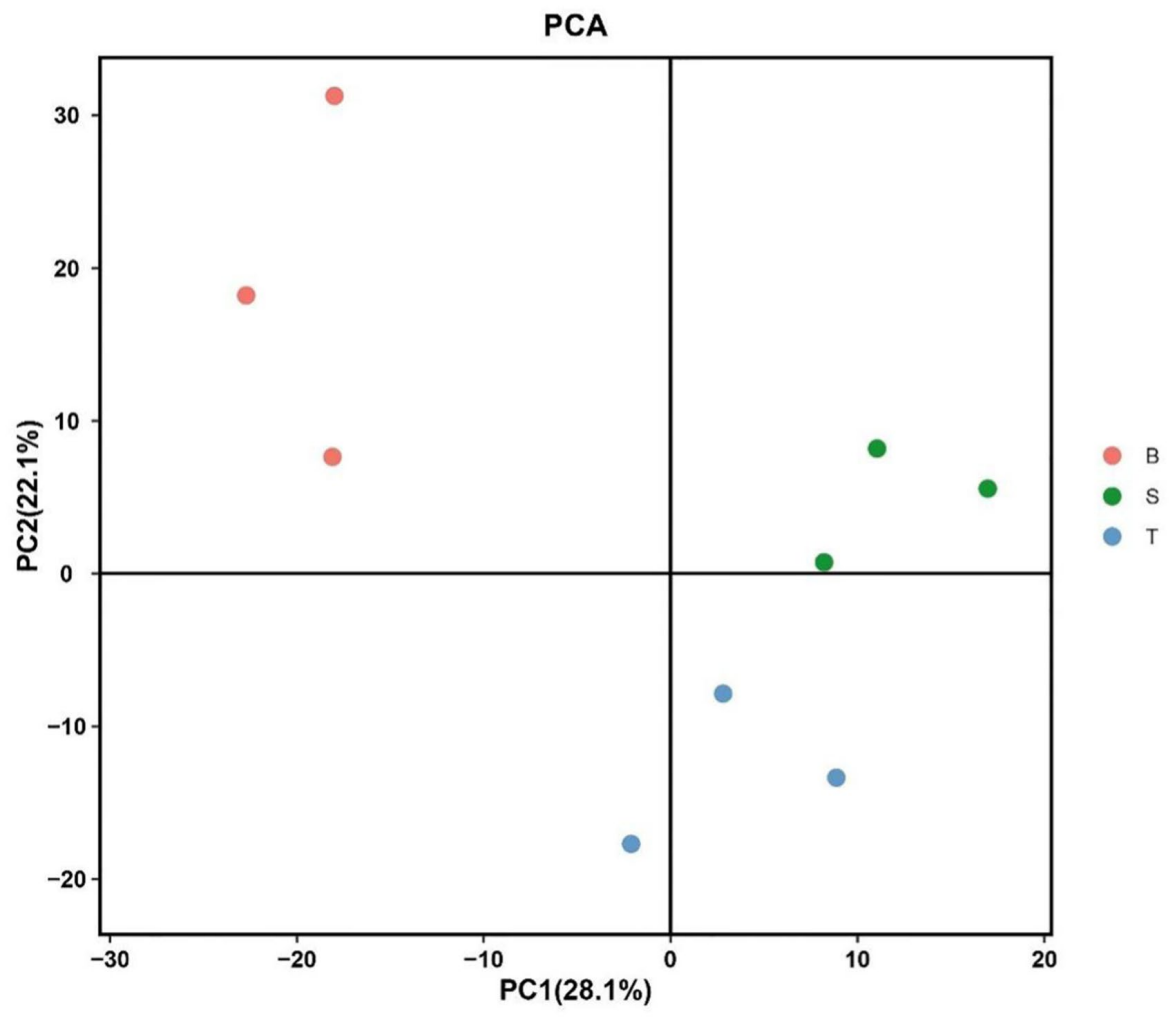
Principal component analysis (PCA) score plot of the metabolomes from three varieties of *P. pubescens.* The plot displays the distribution of samples based on their overall metabolic profiles. S, T, and B represent the varieties “yi ke song”, “tie ba qing”, and “bing he”, respectively. Each point represents an individual biological replicate (*n* = 3 per group). The percentage of total variance explained by each principal component (PC) is indicated in parentheses on the axes.

The data of each group were first analyzed using unsupervised principal component analysis PCA (18). PCA is a dimensionality-reduction technique used to visualize the overall clustering and inherent variation in complex datasets. It simplifies the data by creating new components that capture the maximum variance, allowing us to see whether samples from the same group cluster together and how different groups separate from each other. The PCA model can be used to grasp the metabolite data of different varieties of *P. pubescens* as a whole, especially to find and eliminate abnormal samples and improve the accuracy of subsequent models. The metabolite PCA scores of the different varieties are shown in Figure 3. The results show that the point clouds of the three groups are distributed in different regions, and the distribution areas of the point clouds of each group are relatively close to each other, which indicated that the metabolite composition of each group was less structurally different, with differences in the samples of different fractions, and there was a certain degree of dispersion of the parallel samples in each group, which proved that there were also some individual differences between the samples within the groups. This variation could arise from a combination of genetic, micro-environmental, and subtle developmental differences even within a uniformly managed cultivation plot. And the distributions of Group S and Group T are close in distance. While Group B shows a significant separation from Groups S and T in the sample distribution. There is a trend of grouping between Group S and Group T, which may be due to differences in origin and fruit size.

#### 3.3.2 Partial least squares-discriminant analysis

**Figure 4:**
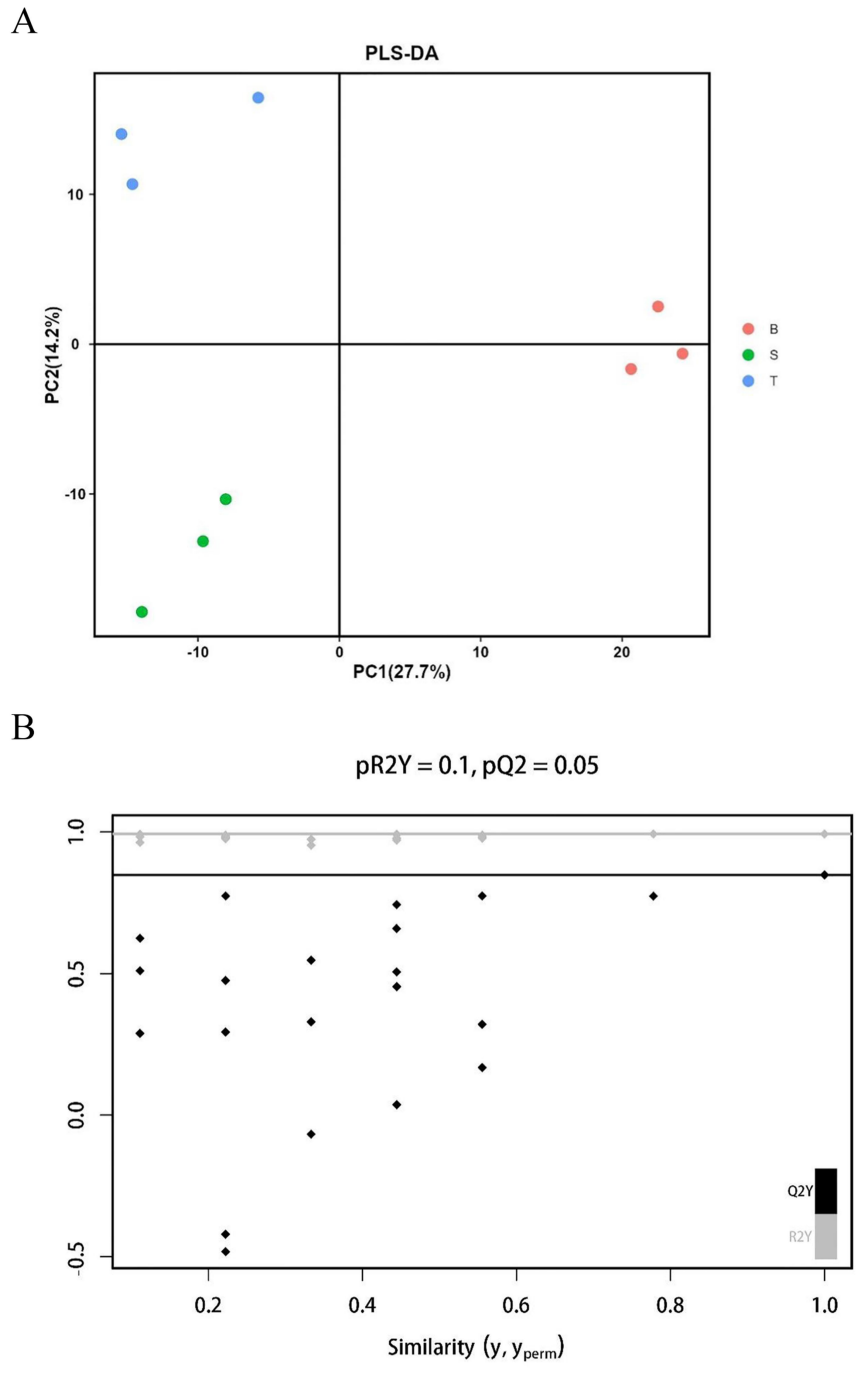
Multivariate statistical analysis and validation of the metabolic profiles from three *P. pubescens* varieties. **(A)** Score plot from the partial least squares-discriminant analysis (PLS-DA). S, T, and B represent the varieties “yi ke song”, “tie ba qing”, and “bing he”, respectively. The plot demonstrates a clear separation among the three varieties (S, T, and B), with each point representing an individual biological replicate (*n* = 3). The model parameters (*R*^2^*Y* = 0.993, *Q*^2^ = 0.848) indicate excellent explanatory and predictive power. **(B)** Permutation test plot (*n* = 200) for validating the PLS-DA model. The model *Q*^2^ points from left to right were much lower than the original *Q*^2^ points at the rightmost end, and the values of *R*^2^ and *Q*^2^ located at the rightmost side were very close to 1, the original model is robust and not overfitted.

Compared with PCA, PLS-DA is a supervised method that actively seeks to find a projection that maximizes the separation between pre-defined sample groups (in this case, the varieties). This makes it particularly powerful for identifying the metabolites that are most responsible for the differences between groups. In this study, the metabolic information of different varieties was further analysed by PLS-DA (19, 20). As can be seen from the PLS-DA score plot (Figure 4A), the three sample groups were very well separated, especially, Group B was better separated by PLS-DA than PCA relative to both Group T and Group S. The model fit parameter (*Q*^2^) and the model discriminant parameter (*R*^2^*Y*) were 0.848 and 0.993 respectively, indicating that the model fit was good and had high predictive power. In essence, a high *Q*^2^ value indicates that the model can reliably predict which variety a sample belongs to based on its metabolic profile. In the replacement test (Figure 4B), the model *Q*^2^ points from left to right were much lower than the original *Q*^2^ points at the rightmost end, and the values of *R*^2^ and *Q*^2^ located at the rightmost side were very close to 1. This indicated that the model predictive ability was high and the model fit was good.

### 3.4 Metabolite identification and analysis of differentially expressed metabolites

**Figure 5:**
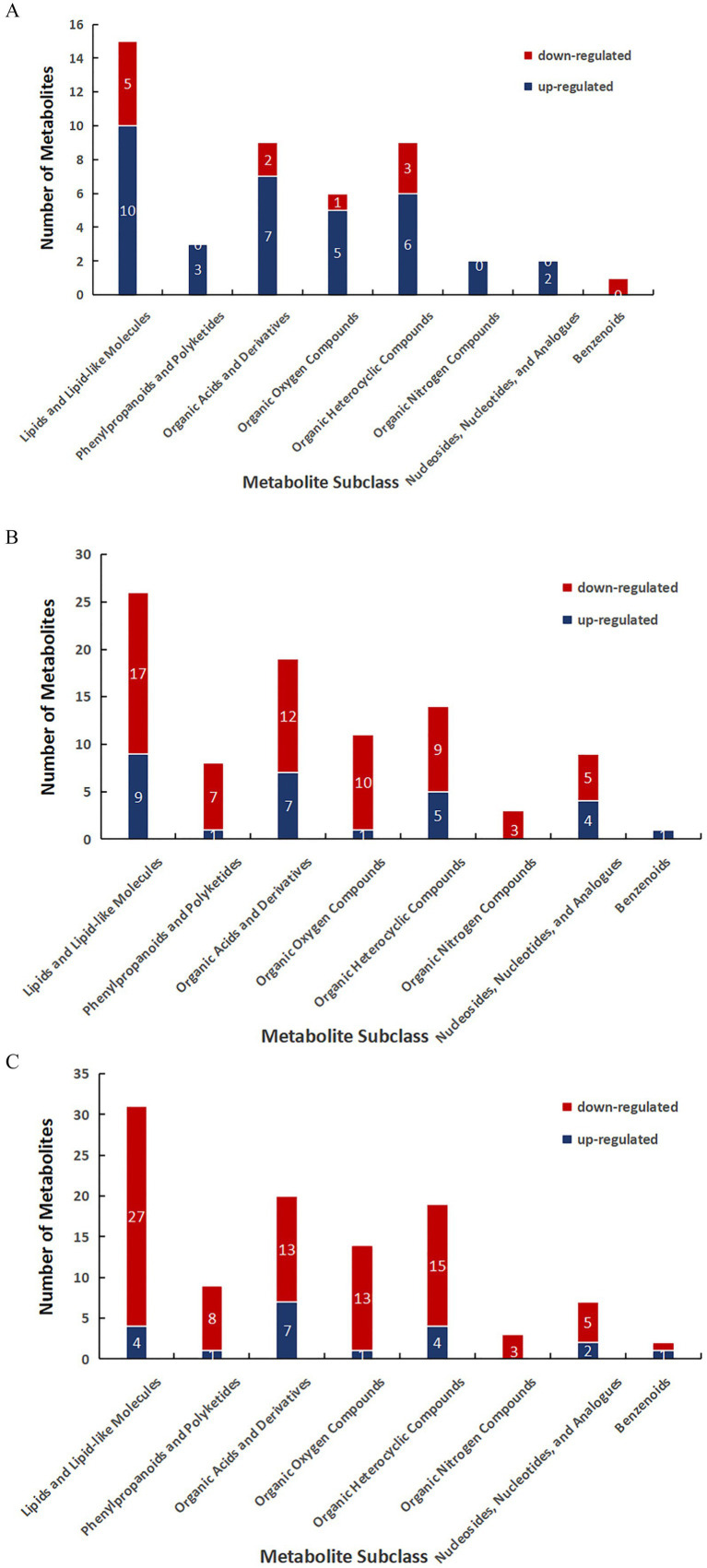
Secondary categories of major differential metabolites **(A–C)**. Categories analysis between variety S and variety T **(A)**. Categories analysis between variety B and variety T **(B)**. Categories analysis between variety B and variety S **(C)**. The *x*-axis represents the subclasses of metabolites, and the y-axis shows the number of differentially expressed metabolites (DEMs). Upregulated metabolites are shown in blue, and downregulated metabolites are indicated in red.

**Figure 6:**
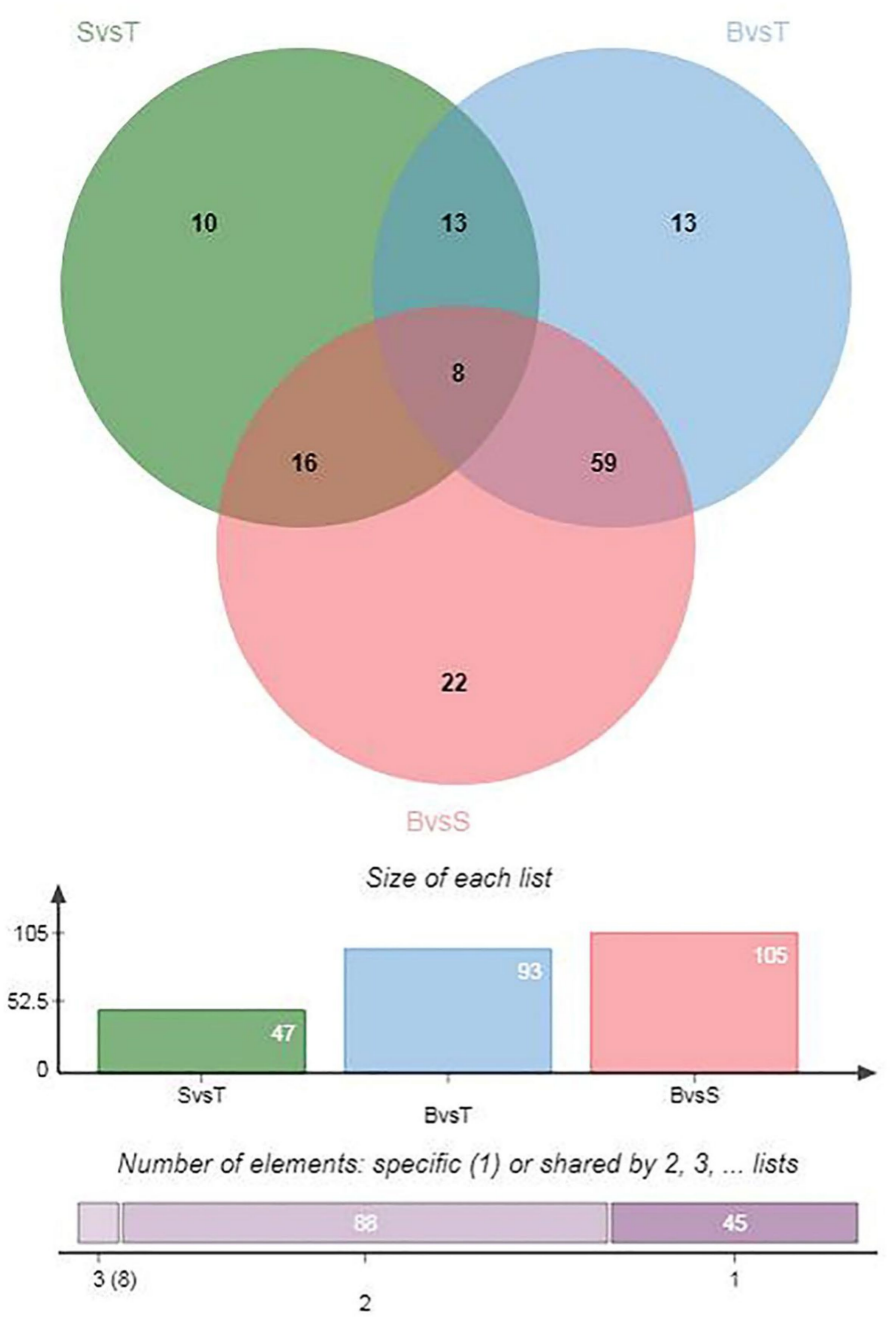
Venn diagram of differential metabolites across pairwise comparisons of the three *P. pubescens* varieties. S, T, and B represent the varieties “yi ke song”, “tie ba qing”, and “bing he”, respectively. The diagram visualizes the number of unique and shared differential metabolites identified from the comparisons of variety B vs. S (BvsS), variety B vs. T (BvsT), and variety S vs. T (SvsT). The overlapping regions indicate common differential metabolites. A total of eight core differential metabolites were common to all three comparisons (central overlap), highlighting a conserved metabolic signature across varieties. The large overlap (67 metabolites) between the BvsS and BvsT comparisons underscores that the metabolic profile of the large-fruited variety B is distinctly different from both small-fruited varieties S and T.

Classification was performed according to the SMILES structural formula, and then the metabolite classification was performed using the analysis program ClassyFire to finally obtain the number of differential metabolites between groups of three different varieties of *P. pubescens* (21), A total of 1,229 metabolites were detected in the combined positive and negative modes of the three varieties by screening for metabolites, variety S versus variety T, the analysis obtained 47 differential metabolites in positive and negative ion mode, of which 35 were up-regulated and 12 were down-regulated; variety S versus variety T, the analysis obtained 93 differential metabolites in positive and negative ion mode, of which 28 were up-regulated and 65 were down-regulated; variety B versus variety S, the analysis obtained 105 differential metabolites in positive and negative ion mode, of which 20 were up-regulated and 85 were down-regulated. In the three comparative varieties, the categories of differential metabolites were consistent, with secondary classifications mainly including lipids and lipid-like molecules, phenylpropanoids and polyketides, organic acids and derivatives, organooxygen compounds, organoheterocyclic compounds, organonitrogen compounds, nucleosides, nucleotides and analogues, and benzene, and substituted derivatives. There were more down-regulated differential metabolites than up-regulated differential metabolites in both variety B samples versus the other two differential comparisons varieties (Figure 5). The Venn analysis of the number of differential metabolites among the three comparison varieties is shown in Figure 6, which revealed that the two combinations of BvsS and BvsT had the most number of identical differential metabolites, 67 in total, while the combination of SvsT and BvsT had 21 identical differential metabolites, and the combination of SvsT and BvsS had 24 identical differential metabolites. Core differential metabolites were screened among the three varieties. The Venn diagram shows eight common differential metabolites that can distinguish three different varieties of *P. pubescens* (Table 1).

**Table 1:** Shared differential metabolites among the three comparative groups.

Name	Molecular formula	Retention (min)	VIP value
13(S)-HPOT	C_18_H_30_O_4_	11.612	1.0780070
19-Hydroxy-4-androstene-3,17-dione	C_19_H_26_O_3_	11.580	1.2525815
3beta-Hydroxypregn-5-en-20-one sulfate	C_21_H_32_O_5_S	9.417	1.3198921
Acetylcholine	C_7_H_16_NO_2_	1.005	1.5111790
Nicotinuric acid	C_8_H_8_N_2_O_3_	4.458	1.5500497
S-(Hydroxyphenylacetothiohydroximoyl)-L-cysteine	C_11_H_14_N_2_O_4_S	2.707	1.3454218
Sphingosine 1-phosphate	C_18_H_38_NO_5_P	10.013	1.1118586
beta-D-Glc-(1- > 4)-alpha-L-Rha-(1- > 3)-D-Glc	C_18_H_32_O_15_	3.667	1.7591158

### 3.5 Heat map analysis of differential metabolites

**Figure 7:**
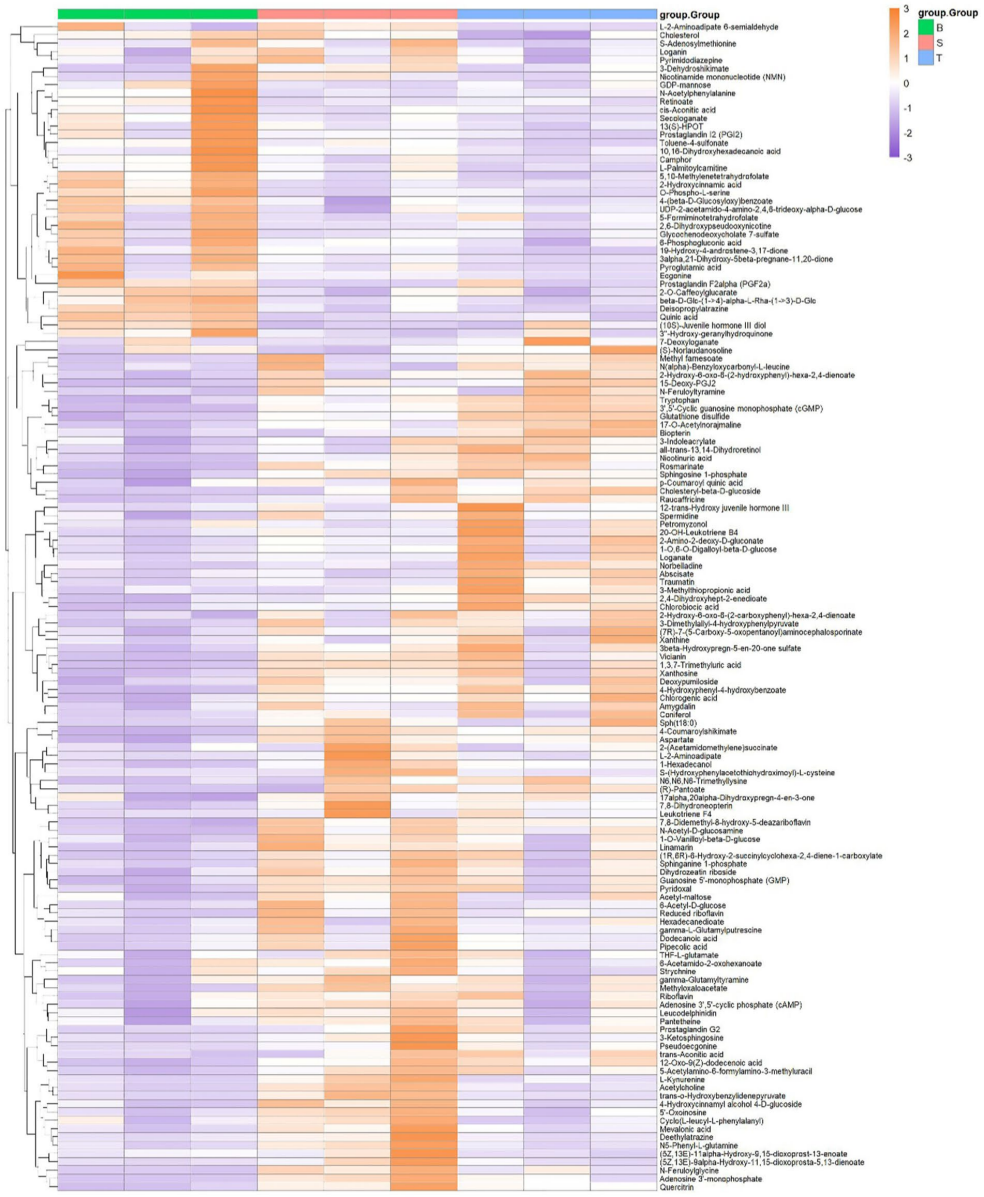
Hierarchical clustering heatmap of differential metabolites among three varieties of *P. pubescens*, where columns represent samples and rows represent metabolites. S, T, and B represent the varieties “yi ke song”, “tie ba qing”, and “bing he”, respectively. Different colors correspond to normalized values of relative metabolite content after standardization. The color gradient in the figure ranges from purple to orange, with purple and orange indicating downregulated and upregulated metabolites in the two sample groups, respectively. Darker purple signifies a greater degree of downregulation, while darker orange indicates a greater degree of upregulation. The annotation bar above the heatmap corresponds to the sample groups.

Cluster analysis was used to determine the metabolic patterns of the metabolites of different varieties of *P. pubescens* under different experimental conditions. Hierarchical cluster analysis was done using the relative values of metabolites as metabolic levels and the results are represented in Figure 7. The colors in the heat map of differential metabolites ranged from purple to orange, and purple and orange are used to indicate down-regulated and up-regulated metabolites in the two sets of samples, respectively. Darker purple signifies a greater degree of downregulation, while darker orange indicates a greater degree of upregulation. Among the three groups of samples, a total of 50 down-regulated differential metabolites were found in large-fruited variety B relative to the same in small-fruited varieties S and T, while large-fruited variety B had a total of 17 upregulated differential metabolites compared to small-fruited varieties S and T.

### 3.6 Analysis of differentially expressed metabolite pathways

**Figure 8:**
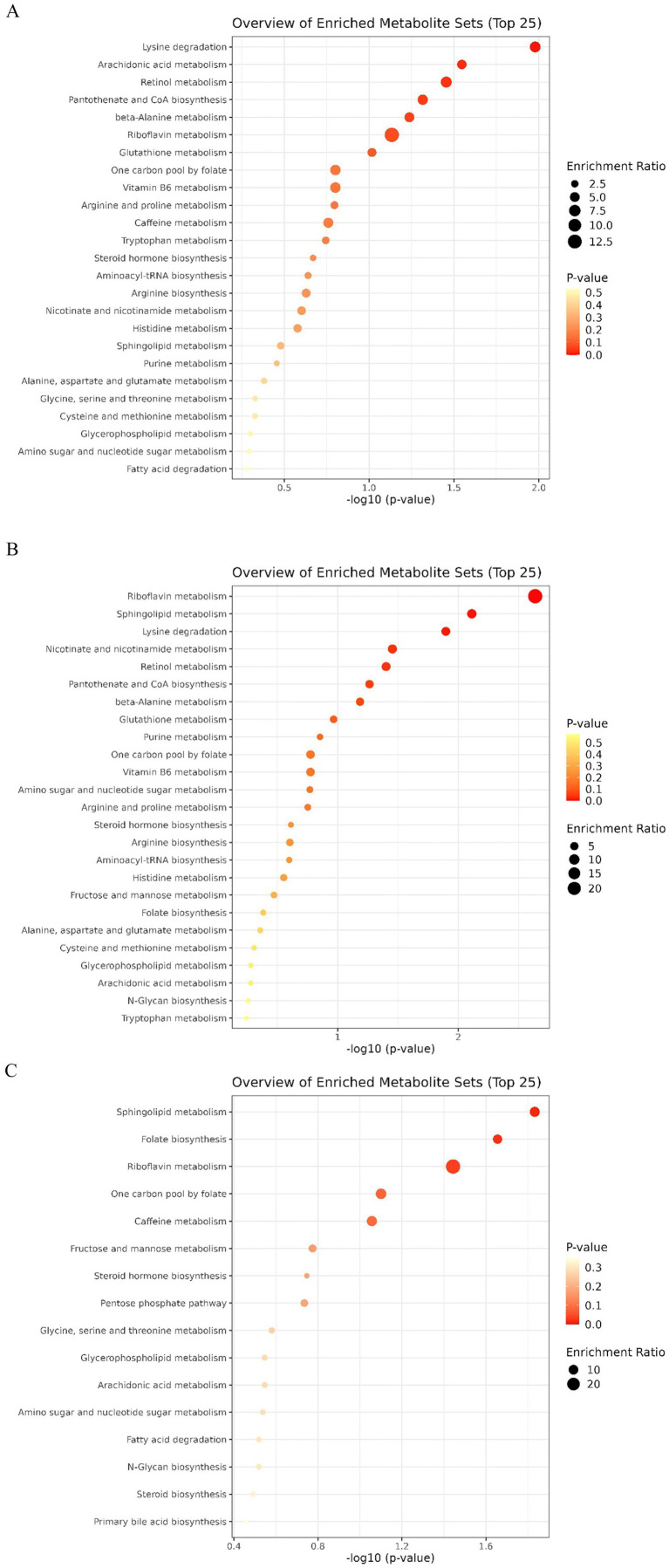
Pathway enrichment analysis of differential metabolites from pairwise comparisons of *P. pubescens* varieties. Bubble plots display the results of KEGG pathway enrichment analysis for comparisons between: **(A)** variety B and S (BvsS), **(B)** variety B and T (BvsT), and **(C)** variety S and T (SvsT). The bubble color represents the significance level of the enrichment [−log10 (*p*-value)], with a redder color indicating a more significant enrichment. The bubble size represents the pathway impact value from topological analysis, with a larger size indicating a greater influence of the pathway in the metabolic network.

Based on the KEGG metabolic pathway MetPA database was used for metabolic pathway concentration and topological analysis,and the possible metabolic pathways affected by different varieties of *P. pubescens* were identified, and then analyzing the metabolic pathways of metabolites. Pathway enrichment analysis showed that the enriched pathways were mainly in metabolism and biosynthesis with a total of 34 metabolic pathways enriched in the three sets of comparisons, as shown in the bubble diagram (Figure 8). The horizontal coordinate where the bubble is located and the size of the bubble represent the influence value, the larger the bubble, the more significant the path is; the vertical coordinate where the bubble is located and the color of the bubble represent the *p*-value for the enrichment analysis of the influence value, the redder the bubble, the closer the *p*-value is to 0, and therefore the more significant the enrichment is. The key pathways in which important differentially expressed metabolites were found to be mainly involved in the comparison of the three varieties were riboflavin metabolism, lysine degradation, sphingolipid metabolism, sphingolipid metabolism, nicotinate, and nicotinamide metabolism.

## 4 Discussion

This study is based on untargeted metabolomics, primarily utilizing ultra-high-performance liquid chromatography (UPLC) and high-resolution mass spectrometry (HRMS) to conduct a metabolomic analysis of *P. pubescens*. The research evaluates the differences among three different varieties and performs extensive bioactivity screening of the metabolites in *P. pubescens*.

Analysis of differential metabolites showed that at the tertiary metabolic level, the major classes of differential metabolites were flavonoids, steroids, phenylpropanoids, amino acids, and alkaloids, aligning with prior findings on the key chemical composition of *P. pubescens* (4, 5). The Venn analysis revealed that the two combinations of BvsS and BvsT had the most number of identical differential metabolites, 67 in total, suggesting that the metabolite differences between variety B(large-fruited variety) and variety S and T(small-fruited varieties) are significant, whereas metabolite differences between varieties S and T both being small-fruited varieties were relatively small, this indicates that the size of the fruit has a significant influence on the chemical composition of *P. pubescens*. Core differential metabolites were screened among the three groups. The Venn diagram shows eight common differential metabolites that can distinguish three different varieties of *P. pubescens*. These shared metabolites mainly include fatty acids, steroids, amino acids, and alkaloids.

A total of 50 down-regulated differential metabolites were found in large-fruited variety B relative to the same in small-fruited varieties S and T, including steroids steroidal such as cholesteryl-beta-d-glucoside, 19-hydroxy-4-androstene-3, 17-dione, and retinoate, amino acids such as N-feruloyltyramine, cyclo(L-leucyl-L-phenylalanyl), pantetheine, and tryptophan; flavonoids such as quercetin as well as phenylpropanoids such as 2-hydroxycinnamic acid, hydroxycoumarin, and so on. Of particular note, quercetin was not detectable in the large-fruited variety B, indicating an extremely low relative content or potential absence that distinguishes it from the small-fruited varieties. This stark contrast suggests that the biosynthetic pathway or regulation of this particular flavonoid is impaired or significantly down-regulated in the large-fruited phenotype. Quercetin is a type of flavonoid that has been widely used and studied as an antioxidant (22), it can decrease the adverse effects related to chemotherapy owing to their antioxidation, antimutagenesis, anti-inflammation, and immunomodu lation (23, 24), many studies have documented that flavonoid chemosynthesis is very effective in cancer chemoprevention and chemotherapy with fewer side effects (25). Research indicates that quercetin is a unique compound due to its potential to combat cancer-related diseases through a multi-target approach (26, 27). Moreover, quercetin has been shown to inhibit the release of P-glycoprotein in the MCF-7 cell line and enhance the *in vitro* anticancer activity of doxorubicin in breast cancer cell lines (28). The high quercetin content in small-fruited varieties may enhance their antitumor potential, which warrants further validation through activity assays. The relative contents of all phenylpropanoid metabolites in large-fruited variety B were lower than those in small-fruited varieties S and T, phenylpropanoid is reported to possess anti-inflammatory, antiviral, anti-allergenic, antibacterial, and antioxidant properties, which are beneficial to human health, it also inhibits carcinogenesis and reduce the risk of diabetes and heart disease (29). Seventeen differential metabolites were present in higher relative amounts in large-fruited variety B than in small-fruited varieties S and T, including amino acids such as aspartate, L-2-aminoadipate, N_6_, N_6_, N_6_-trimethyllysine, 2-smino-2-deoxy-D-gluconate and pipecolic acid, phenolics such as coniferol, alkaloids such as 17-O-acetylnorajmaline, and vitamins such as (R)-pantoate, riboflavin and pyridoxal. Amino acids play a wide variety of physiological roles in plants, including promoting growth and development, and offering phenylalanine as an intermediate in the biosynthesis of most plant phenolics (12). Pipecolic acid, a frequent metabolite in the body, play a significant role in the development of a variety of illnesses, including obesity and arabidopsis immunity, and can also slow down diabetes retinopathy (30). Tryptophan is an essential amino acid, which plays a role in immune homeostasis, as local tryptophan catabolism impairs T-lymphocyte mediated immunity (31). Aspartate may be a limiting metabolite for tumor growth, and aspartate availability could be targeted for cancer therapy (32). From the results of the study, it was again demonstrated that both the type and content of metabolites differed among the different varieties of *P. pubescens* especially those with significant differences in fruit size. Quercetin and phenylpropanoids absence in variety B strongly suggests a diminished potential for these health-promoting activities compared to the small-fruited varieties S and T, where it was abundant. This positions the small-fruited varieties as superior raw materials for developing health products aimed at mitigating oxidative stress or inflammation. Conversely, the elevated levels of riboflavin (Vitamin B_2_) in the large fruits are noteworthy from a basic nutritional standpoint. Variety B might be promoted as a good dietary source of this essential vitamin, catering to a different nutritional niche.

Important differentially expressed metabolites play a crucial role in key pathways. The riboflavin metabolism pathway showed overlapping patterns among the three comparison groups, with riboflavin being identified as a differentially expressed metabolite in all groups. Riboflavin (RF), also known as Vitamin B_2_, is one of the essential micronutrients for the human body and has been ranked by the World Health Organization (WHO) as one of the six main indicators for assessing human growth (33). Lack of riboflavin can affect the body’s biological oxidation–reduction reaction, cause metabolic disorders, and lead to a series of diseases, and in severe cases, even death (34). In our study, riboflavin consistently emerged as a significant and characteristic differential metabolite across all comparisons. This strong association suggests that riboflavin has the potential to serve as a metabolic marker for identifing and differentiating *P. pubescens* varieties.

## 5 Conclusion

In this study, three varieties (one large and two small) of *P. pubescens*, i.e., “tie ba qing”, “yi ke song”, and “bing he”, were compared based on UPLC-ESI-MS/MS metabolomics, which mainly consisted of ultra-high performance liquid chromatography and high-resolution mass spectrometry. The results of the analyses showed that (1) a total of 50 down-regulated differential metabolites, including steroids, amino acids, flavonoids, and phenylpropanoids, were down-regulated in large-fruited variety B relative to the same in small-fruited varieties S and T. This suggests that for most of the differential metabolite contents small-fruited varieties are higher than large-fruited variety. (2) The flavonoid quercetin was not detected in the samples of the large-fruited variety B, highlighting a major compositional difference. The relative contents of all phenylpropanoid metabolites in large-fruited variety B were lower than those in small-fruited varieties S and T, and the antioxidant and anti-inflammatory functions of samples of the S and T varieties were better. (3) The same upregulated differential metabolites in large-fruited variety B relative to small-fruited varieties S and T totaled 17, with relatively high relative contents of the phenolic substance coniferol and the functional vitamin class riboflavin. (4) Metabolic pathway analyses revealed that riboflavin was a consistently enriched differential metabolite across all three comparisons, identifying it as a robust and characteristic metabolic feature for distinguishing *P. pubescens* varieties in this study. The above results suggest that different varieties of *P. pubescens* especially those with large variation in morphology and size may affect their phytochemical composition. Our findings provide new insights into the phytochemical composition of different varieties of *P. pubescens*. This study can help to understand the quality characteristics and metabolic mechanisms among the varieties with different fruit forms of *P. pubescens*. Building upon the findings of this study, several promising future research directions emerge. Firstly, the functional implications of the distinct metabolic profiles, particularly the high quercetin and phenylpropanoid content in small-fruited varieties, should be validated through *in vitro* and *in vivo* assays to confirm their superior antioxidant and potential antitumor activities. Secondly, the potential of riboflavin and the other core differential metabolites as reliable markers for variety identification and quality control should be assessed in a larger and more diverse set of *P. pubescens* samples, including those from different geographical origins and harvest seasons. Finally, investigating the genetic and molecular mechanisms underlying these metabolic differences, especially those related to fruit size, would be a crucial next step. Such research could identify key genes regulating the biosynthesis of important metabolites like quercetin, opening avenues for molecular breeding programs aimed at enhancing the nutritional and medicinal value of *P. pubescens*.

## Data Availability

The original contributions presented in the study are included in the article/supplementary material, further inquiries can be directed to the corresponding authors.
